# Analysis of conditioning with dexmedetomidine under endothelial dysfunction in isolated perfused hearts

**DOI:** 10.3892/br.2025.2014

**Published:** 2025-06-11

**Authors:** Sophia De Luca-Rohner, André Heinen, Martin Stroethoff, Annika Raupach

**Affiliations:** 1 Department of Anaesthesiology, Medical Faculty and University Hospital Düsseldorf, Heinrich-Heine-University Düsseldorf, Düsseldorf D-40225, Germany; 2 Institute for Cardiovascular Physiology, Medical Faculty and University Hospital Düsseldorf, Heinrich-Heine University Düsseldorf, Düsseldorf D-40225, Germany; 3 CARID, Cardiovascular Research Institute Düsseldorf, University Hospital Düsseldorf, Heinrich-Heine University Düsseldorf, Düsseldorf D-40225, Germany

**Keywords:** endothelial dysfunction, dexmedetomidine, conditioning, cardioprotection, ischemia/reperfusion

## Abstract

Cardioprotective strategies such as pharmacological conditioning have not yet successfully undergone bench-to-bedside transfer, which is probably due to inhibition of cardioprotection by comorbidities or associated pathological changes. Endothelial dysfunction (ED) is closely associated with most cardiovascular diseases and their typical comorbidities. Therefore, cardioprotective strategies should be examined under ED. It was previously demonstrated that dexmedetomidine (DEX) maintains its cardioprotective properties against ischemia/reperfusion (I/R) injury under hyperglycaemia in the setting of pre-but not postconditioning, using a constant pressure Langendorff system. Under ED, cardioprotection by DEX preconditioning is also maintained using a constant flow mode, whereas this has not yet been investigated for postconditioning. Because DEX has vasoconstrictive properties, different haemodynamic conditions might influence the cardioprotective potential of DEX. Therefore, it was investigated whether pre- and postconditioning protocols with DEX used in constant pressure mode are transferable to constant flow mode and whether the cardioprotective effect of DEX is maintained under ED. The cardioprotective effect against I/R injury of pre- and post-treatment with 3 nM DEX on isolated-perfused hearts of male Wistar rats was analysed in constant flow mode. ED was induced by perfusion with Krebs-Henseleit buffer containing 60 mM KCl. Heart function was assessed via pressure measurements in the left ventricle (LV) and infarct size (IS) via triphenyltetrazolium-chloride staining. In constant flow mode, pre- and post-treatment with DEX has no effect on IS and heart function compared with hearts treated with vehicle both under ED and under physiological conditions. In DEX pre-treated hearts under ED, LV developed pressure is increased and contractility is improved after 60 min of reperfusion. Pre- and post-treatment with DEX is not cardioprotective with the protocol used, while DEX pre-treatment under ED has improving effects on heart function. Different hemodynamic conditions may modulate the cardioprotective properties of DEX, possibly due to its vasoconstrictive properties.

## Introduction

Over the last decades, a plethora of experimental studies and clinical trials investigating cardioprotective strategies such as ischemic or pharmacological conditioning to protect against or minimize the extent of ischemia/reperfusion (I/R) injury have been published (1). The demand for this kind of protection is high, because I/R injury such as myocardial infarction are among the leading causes of death worldwide (2). Unfortunately, attempts to transfer cardioprotective strategies from bench to bedside have not been successful thus far (1). This appears to be due to the presence of confounding factors, including comorbidities such as diabetes mellitus or associated pathological changes which inhibit cardioprotective mechanisms, for example in signal transduction (3). Therefore, cardioprotective strategies have to be investigated not only under physiological but also under pathological conditions.

Endothelial dysfunction (ED) promotes or is closely associated with most cardiovascular diseases (CVD) and their typical comorbidities such as diabetes mellitus (4). In the presence of ED, insufficient bioavailability of nitric oxide (NO) in the vascular system causes an imbalance in vascular tone with reduced endothelium-dependent relaxation in response to vasodilatory stimuli (5,6). In light of the high number of patients affected by both CVD and ED and the potential of a loss of efficacy of cardioprotective strategies under these pathological conditions, cardioprotective strategies should be examined under ED.

Dexmedetomidine (DEX), a highly selective α2-adrenergic receptor agonist, has cardioprotective properties against I/R injury regardless of whether DEX is used in a setting of pre- or postconditioning (7,8). Furthermore, this protection appears to be effective even under pathological conditions, as He *et al* (9) have shown that cardioprotection against I/R injury by preconditioning with DEX can be maintained under ED using the *in vitro* model of an isolated perfused rat heart. In line with this, it has been previously demonstrated by the authors that protection by preconditioning with DEX persists even under hyperglycaemia, a further pathological condition associated with CVD (10). However, this was not the case for protection induced by postconditioning with DEX. Whether the protective effect of DEX postconditioning is maintained under ED is, to the best of our knowledge, not known.

In addition to its cardioprotective properties, DEX also has an influence on the hemodynamic response of the vasculature. Infusion of DEX induces an initial vasoconstriction by activation of postsynaptic α2B adrenoreceptors (AR), which in turn is reversed by activation of α2A AR in the central nervous system mediated by a reduction in blood pressure and heart rate (11). As isolated-perfused hearts lack a neural connection, only vasoconstriction is caused by DEX, potentially resulting in increased vascular tone and higher perfusion pressure. This is particularly relevant in the context of ED, where impaired NO bioavailability leads to an inability to counteract vasoconstriction effectively. Thus, DEX may exacerbate vascular damage by further increasing resistance and pressure in the coronary vessels, which can negatively influence its cardioprotective effects against I/R injury. In our previous studies investigating conditioning with DEX, a constant pressure system was used, as has been recommended for the investigation of cardioprotective strategies against I/R injury (7,8,12). In constant pressure mode, the vascular system of the heart can react individually to the perfusion pressure through vasomotion and thus during phases of high pressure induce dilation to reduce vascular damage. In this mode, the perfusion flow is also regulated via the vascular resistance. By contrast, in constant flow mode, which was used by He *et al* (9) to study preconditioning with DEX under ED, a pre-specified pump flow rate is maintained against any vascular resistance, leading to higher pressures and therefore potentially greater vascular damage during vasoconstriction. He *et al* (9) perfused a buffer containing high amounts of potassium for ED induction, whereas for successful damage of endothelial function by such solutions, a constant flow mode is mandatorily (13). In order to be able to use this protocol as well, the concept of treatment duration and concentration for conditioning with DEX from our former studies with a constant pressure system was transferred to the constant flow mode (7). However, due to the vasoconstrictive properties of DEX, the choice of perfusion mode could influence the cardioprotective effectivity of DEX. When DEX is used in constant pressure mode, the vasoconstrictive effect of DEX could increase resistance and elevate perfusion pressures, resulting in a reduction of perfusion flow. Otherwise, in a flow-constant system, the combination of a lack of adaptability to an increased vascular pressure via a reduction in perfusion flow and DEX-induced vasoconstriction can lead to increased shear stress, which in turn can be responsible for severe damage to the vessels and thus also to the tissue.

The aim of the present study was to investigate: i) if 5 min pre-treatment with 3 nM DEX has cardioprotective effects in the setting of ED in a constant flow Langendorff system; ii) if ED influences effects of post-treatment with DEX and iii) if different experimental modes (constant pressure vs. constant flow) modulate protective effects.

## Materials and methods

### Animals

All experiments were performed in accordance with the Guide for the Care and Use of Laboratory Animals, published by the U.S. National Institute of Health (NIH publication no. 85-23, revised 1996), after approval (approval no. O27/12, 10.05.2012) by the Center for Animal Experiments and Scientific Animal Welfare (ZETT) Heinrich-Heine University (Düsseldorf, Germany). The animals were under the care of the ZETT. The rats had *ad libitum* access to food and water during a 12/12-h light-dark cycle at a temperature of 22±3˚C and a relative humidity of 55±10%. The animals' health and behaviour were monitored daily. Humane endpoints were not defined, as this *in vitro* study was based on organ removal from healthy animals following euthanasia by decapitation under sedation. In the case of a change of location, the animals were given sufficient acclimatization time to minimize stress caused by transport and the new environment. The cages contained sufficient bedding and enrichment for hiding to meet the rats' need for safety. Every effort was made to minimize the number and suffering of the animals in the present study. A total of 42 animals were randomized into six groups. No animal had to be euthanized or was found dead prior to the start of the experiments. Two animals were excluded from analysis because their hearts did not fulfil the minimum criterion for the left ventricular developed pressure of 80 mmHg at the end of the adaption phase in the Langendorff system. Therefore, a total of 40 animals were included in the analysis.

### Langendorff system

Male Wistar rats aged 2-3 months were randomized into the respective groups. Prior to decapitation for euthanasia, the animals were sedated by intraperitoneal injection of 100 mg/kg body weight sodium-pentobarbital (Narcoren; Boehringer Ingelheim) to ensure pain- and stress-free euthanasia. Agglutination was prevented by injection of 3,330 IU/kg heparin sodium (Braun SE). Following confirmation of an adequate depth of sedation, indicated by the absence of the toe pinch reflex (after ~5 min), the animals were decapitated with a guillotine. After chest opening, hearts were isolated and mounted on a constant flow Langendorff system (12 ml/min) and perfused with a modified Krebs-Henseleit buffer (KHB; 118 mM NaCl, 4.7 mM KCl, 1.2 mM MgSO_4_, 1.2 mM KH_2_PO_4_, 24.9 mM NaHCO_3_, 2.5 mM CaCl_2_, 1 mM lactate, 0.5 mM EDTA; and 11 mM glucose at 37˚C). For the induction of global ischemia, perfusion with KHB was interrupted and the heart was then immersed in KHB aerated with nitrogen. Reperfusion was induced by restoring the heart perfusion with KHB while the ischemic buffer bath around the heart was removed. For treatment with DEX, a solution of 300 nM dexmedetomidine hydrochloride (cat. no. SML0956; Merck KGaA) dissolved in KHB was introduced into the perfusate via a syringe driver with 1% of the flow rate of the Langendorff system to treat the heart with an effective concentration of 3 nM DEX. For the induction of ED, hearts were perfused with KHB containing 60 mM KCl (K+) for 10 min. In this buffer variant, the amount of NaCl was reduced from 116 to 63 mM to maintain equal osmotic conditions. The buffer composition and treatment duration was adapted from a protocol published by He *et al* (9). In their study, successful ED induction was demonstrated by the inability of K^+^-treated hearts to respond adequately with vasodilation to the application of the vasodilator histamine. The perfusion with K^+^ was implemented via a separate circuit in the Langendorff system to ensure an exact duration of the perfusion and to avoid buffer mixing. A water-filled balloon placed in the left ventricle (LV) was used for continuous LV pressure measurements, which were digitized using an analogue to digital converter (PowerLab/8SP, ADInstruments Pty Ltd.) at a sampling rate of 500 Hz and recorded on a Personal Computer using Labchart 8.0 for Windows (ADInstruments Pty Ltd.). During the adaptation phase, the left ventricular diastolic pressure (LVP min) was set at 3 to 5 mmHg. The following hemodynamic variables were determined: heart rate, LVP min, left ventricular systolic pressure (LVP max), left ventricular developed pressure (LVDP=LVP max-LVP min), maximum rate of pressure change in the LV (dP/dt max), and minimum rate of pressure change in the LV (dP/dt min). A pressure transducer connected near the heart measured coronary perfusion pressure (CPP).

### Experimental setup

**Figure 1:**
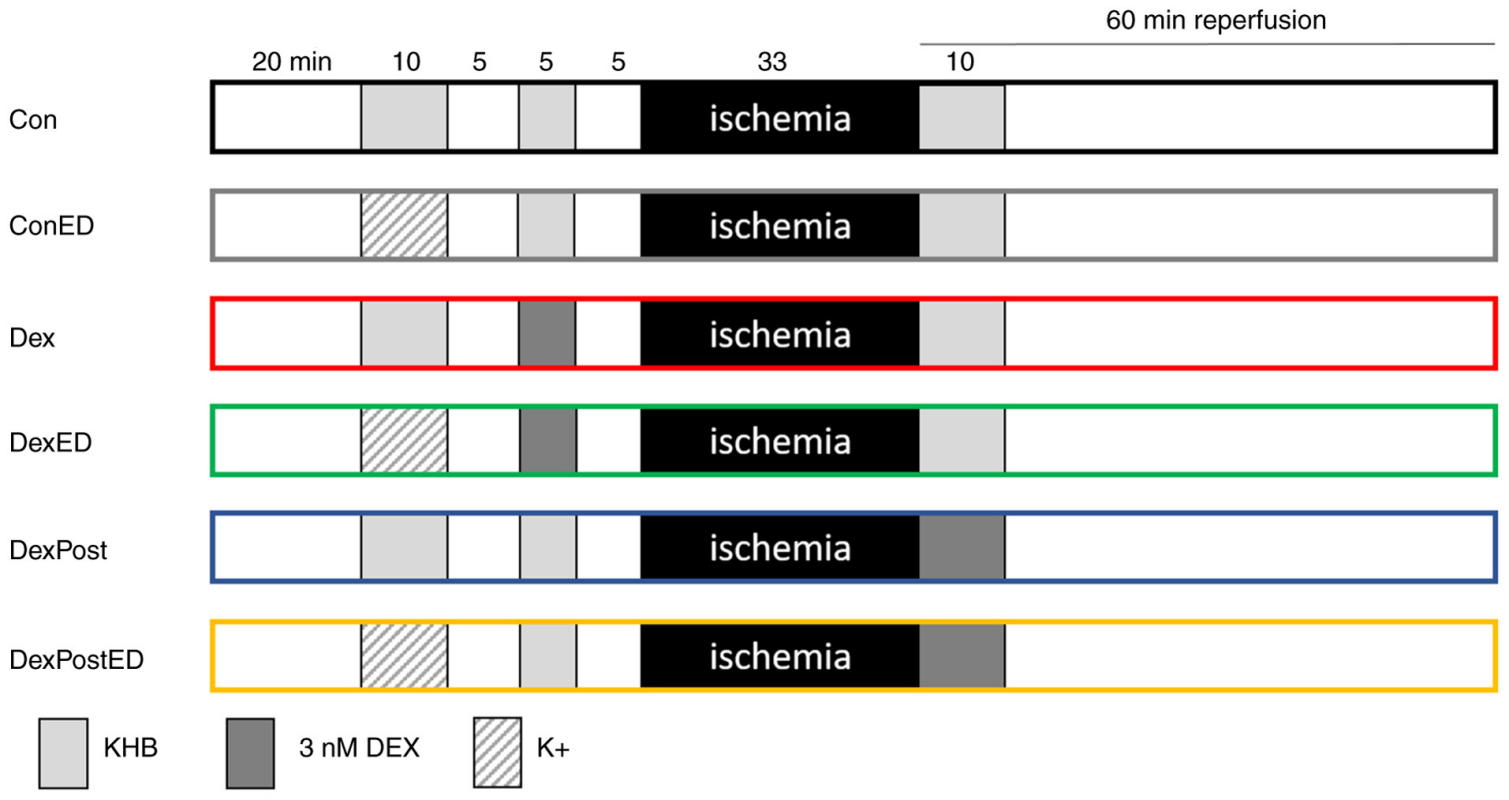
Experimental protocol: Hearts of male Wistar rats are pre-treated with 3 nM DEX or KHB as vehicle (control; Con) for 5 min. For post-treatment, hearts are treated for 10 min with 3 nM DEX with onset of reperfusion. ED is induced by perfusion with KHB containing 60 mM KCl (K^+^); as a control condition, perfusion with normal KHB is performed. DEX, dexmedetomidine; KHB, Krebs-Henseleit-buffer; ED, endothelial dysfunction.

All hearts underwent the following protocol: 20 min adaption phase, 10 min induction of ED, 5 min washout, 5 min first treatment phase (1st TP), 5 min washout, 33 min ischemia, and 60 min reperfusion, including a second treatment phase of 10 min at its beginning (Fig. 1). Hearts were randomized into 6 groups. Hearts in control group Con received only KHB, while those in group ConED were perfused with K^+^ for 10 min to induce ED. In the DEX pre-treatment groups, hearts were treated with 3 nM DEX for 5 min during the first treatment phase after they had been previously perfused with either K^+^ for the induction of ED (DexED) or with KHB as a control (Dex). In a previous study, a pre-treatment duration of 5 min and a concentration of 3 nM DEX had revealed cardioprotective effects on infarct size (7). Increasing the concentration to 30 nM DEX or extending the treatment duration to 10 or 25 min did not improve the extent of infarct size reduction by pre-treatment with DEX. To investigate a potential impact of ED on post-treatment with DEX, hearts were perfused with 3 nM DEX in the second treatment phase for 10 min, which had cardioprotective effects on infarct size in previous studies, whereas shortening the treatment phase to 5 min for post-treatment has not yet been investigated (8,10,14). After 20 min adaption phase, these two groups were perfused with K^+^ for induction of ED (DexPostED) or with KHB for control conditions (DexPost).

### Infarct size determination

To determine infarct size, a staining with 0.75% triphenyl-tetrazolium chloride (cat. no. 37130.02; SERVA Electrophoresis GmbH) was performed on trimmed 1-mm thick heart slices. For quantification, an investigator blinded to group assignment measured the total area of the LV and the infarcted areas using planimetry, from which infarct size was calculated as the infarcted area expressed as a percentage of the LV area (SigmaScan Pro5; Systat Software, Inc.).

### Statistical analysis

A-priori sample size analysis with infarct size chosen as the primary endpoint was performed with G*Power 3.1.9.7(15); all other statistical analyses statistical analyses were performed using GraphPad Prism 10 (Dotmatics). The impact of ED on pre- or post-treatment with DEX was analysed using a one-way analysis of variance (ANOVA) followed by Tukey's multiple comparisons tests. The calculated total sample size needed to detect differences on infarct size between 6 groups was 42 (effect size 0.65, power 80%, α=0.05). For variables measured longitudinally over time, baseline measurements were compared with ensure equivalent starting points. In addition, for comprehensive assessment, measurements received after 60 min of reperfusion were analysed. To analyse vasoconstriction induced by DEX before ischemia, two-way ANOVA (conditioning x endothelial function) at time point 1st TP 4 min on values of LVDP was performed. The level of significance was defined as P<0.05, otherwise the effect was declared not significant. Data are shown as the mean ± standard deviation (SD).

## Results

**Figure 2:**
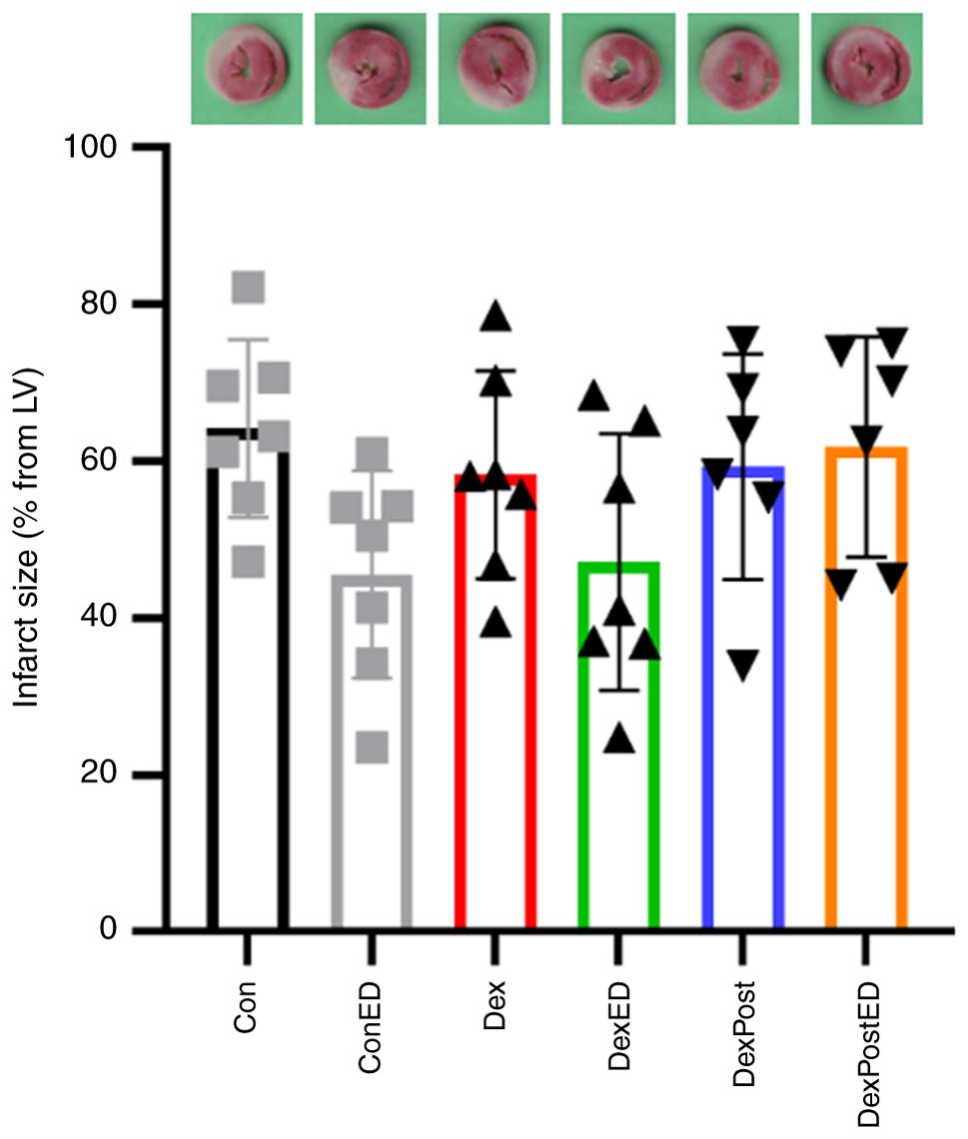
Infarct sizes: Quantification of infarct sizes from hearts pre-treated for 5 min or post-treated (Post) for 10 min with 3 nM DEX or with vehicle Krebs-Henseleit-buffer as control (Con) under healthy conditions or ED. A representative heart slice stained with triphenyltetrazolium chloride is shown for each group. The individual infarct sizes are each shown with one data point. Data are presented as the mean ± SD (n=6-7). One-way ANOVA revealed no significant differences between groups (P>0.05). Dex, dexmedetomidine; ED, endothelial dysfunction; LV, left ventricle.

The cardioprotective effect of 3 nM DEX pre-treatment for 5 min or post-treatment for 10 min under control conditions or ED using a constant flow Langendorff system was examined. As shown in Fig. 2, infarct sizes are not different between groups, meaning neither treatment with DEX nor ED induction has an significant influence on infarct size (Con: 64±11%, ConED: 46±13%, Dex: 58±13%, DexED: 47±16%, DexPost: 59±14%, DexPostED: 62±14%, P=0.0796, F=2.179).

Body weights, wet and dry heart weights are not different between groups (Table I). Peak pressure during ischemia (ischemia peak) is reduced by K^+^ perfusion, independent of pre-treatment with DEX or KHB. Variables of heart function such as heart rate, LVP min, LVP max, LVDP, dP/dt max, dP/dt min, and CPP are not different at baseline between the six groups ensuring equal starting conditions (Fig. S1). After 60 min of reperfusion, the comparison of groups pre-treated with DEX reveals that perfusion with K^+^ increases LVDP. Furthermore, dP/dt max is elevated and dP/dt min is diminished by perfusion with K^+^ in groups pre-treated with DEX.

These data suggest that treatment with 3 nM DEX has no cardioprotective effect, neither on infarct size nor on heart function, in the constant flow Langendorff-system, under physiological conditions or ED, while perfusion with K^+^ in combination with pre-treatment with DEX improves heart function.

## Discussion

In the present study, it was revealed that by using a constant flow Langendorff system, neither pre- nor post-treatment with 3 nM DEX has any cardioprotective effect on infarct size or heart function; this is independent of induction of ED via perfusion with K^+^. In DEX pre-treated hearts, however, an improvement of in heart function after 60 min of reperfusion was detectable under ED. Results from constant pressure mode Langendorff systems are not transferable to constant flow experiments.

In the present study, treatment with 3 nM DEX for 5 min failed to reduce infarct size after 30 min of ischemia and 60 min of reperfusion in a constant flow Langendorff system. By contrast, a similar treatment protocol for preconditioning using a constant pressure system approximately halved the infarct size from 49 to 24% (7). Even lower concentrations of DEX, namely 0.03 and 1 nM DEX, were still able to reduce infarct sizes (reduction by ~10 %), but not to the extent of 3 nM DEX. Interestingly, in a study by He *et al* (9) using a constant flow mode system, a longer pre-treatment duration of 30 min and a slightly higher concentration of 10 nM DEX resulted in an infarct size reduction in hearts of male Sprague-Dawley rats following the same time protocol of I/R injury. Wang *et al* (16) used a comparable protocol to He *et al* (9) and also showed a cardioprotective effect with reduced infarct sizes by treatment with 10 nM DEX for 30 min in a constant flow Langendorff system after a slightly prolonged ischemia time of 40 and 60 min of reperfusion. Thus, both studies underline the protective effect of 30 min treatment with 10 nM DEX in constant flow mode, whereby the small variations in the time protocols for I/R probably have only a minor influence. Therefore, different perfusion conditions appear to modulate cardioprotection by DEX. One possible explanation is that DEX needs a minimal time interval to induce a cardioprotective effect. In constant flow mode, DEX is transported rapidly through the vessels independently of the onset of vasoconstriction, while in constant pressure mode, the beginning of vasoconstriction would reduce the flow, allowing a longer retention time of DEX in the vessels. On one hand, this theory is supported by the observation that in constant pressure mode, a treatment duration of 5 min is sufficient to induce cardioprotection (7). On the other hand, this theory is contradicted by the finding that in these experiments coronary flow, acting as a surrogate for coronary pressure in a constant pressure system, is only slightly reduced by DEX and thus only minimally increased coronary vasoconstriction is shown. In the present study, however, DEX induces vasoconstriction, which is exemplified by the increased CPP after application of DEX (1st TP 4 min; two-way ANOVA, P=0.0018, F=2.502), which persists until onset of ischemia. This could also lead to higher shear stress in constant flow mode, which might in turn lead to more damage to the heart. This could be another explanation for the lack of protection by DEX. Thus, during ischemia, the heart is in ischemic contraction, which means maximal vasoconstriction that slowly starts to abate with the onset of reperfusion. By contrast, in constant flow mode a specific volume is transported per unit of time, regardless of the high vascular pressure at this time point, thus severely damaging the vessels. DEX-induced vasoconstriction could slow down the relaxation or inhibit sufficient relaxation and thus contribute to even greater damage. A further explanation for the lack of cardioprotection by 3 nM DEX in the constant pressure mode could also be differences, for example in sensitivity to DEX, between Wistar (used in our group) and Sprague Dawley rats [used by He *et al* (9) and Wang *et al* (16)], although only very small differences would probably be expected here.

Interestingly, pre-treatment with 3 nM DEX does not lead to cardioprotection in the present study, but increases CPP and thus has a vasoconstrictive effect suggesting that DEX in principle is able to induce its signalling receptors/pathways under the given conditions. However, both functions are mediated via the α2-AR, as the α2-AR antagonist yohimbine can inhibit DEX-induced cardioprotection and also vasoconstriction (17-19). This suggests that successful cardioprotection by DEX requires either a stronger activation of the α2-AR signalling than vasoconstriction or the activation of additional signalling receptors/pathways. One such possible pathway is the binding of DEX to I1-imidazoline receptors (I1R). Yoshikawa *et al* (19) reported that efaroxan, an α2-AR and I1R antagonist, can abolish the reduction in infarct size by preconditioning with DEX in hypertrophied hearts, whereas yohimbine has no effect here. This suggests a role of I1R in the cardioprotective signalling effect of DEX. Whether I1R plays a role in the signalling under the conditions of the present study must be analysed in further studies.

An unexpected finding was the protective effect on heart function in hearts pre-treated with DEX under ED, observed as an increase in LVDP and improved contractility compared with the respective control hearts. In addition, a slight reduction of infarct size under ED is detectable in control and DEX pre-treated groups, whereas this reduction is not observed in the DexPostED group. The protocol for the induction of ED with respect to buffer composition and treatment duration was based on the protocol from the study of He *et al* (9), which has shown no influence on infarct size and heart function by this protocol. The different setup of the Langendorff systems could be responsible for these contradictory results. For the present study, the Langendorff system was modified with a separate circuit for perfusion of K^+^ to ensure exact perfusion durations and to avoid mixing of the buffers, whereas He *et al* (9) did not describe details of the construction of their Langendorff system. Another explanation for the slight protective effect could be the modulation of perfusion of hearts by K^+^. Specifically, compared with hearts perfused with KHB alone, perfusion with K^+^ induces a sustained reduction in LVP max, which, with an unchanged LVP min, results in a decrease in LVDP until the onset of ischemia (Fig. S1A-C). After 60 min of reperfusion, the pressure situation is equal for all groups except an increased LVDP for group DexED, indicating improved cardiac function. A possible explanation for the protection induced by perfusion with K^+^ could be that hearts with lower LVDP before ischemia could reduce their energy demand, which might result in an improved performance in the reperfusion phase. Furthermore, independently of the modulation of hemodynamic variables by K^+^, cardioprotective signalling might be activated. The hypercontraction due to perfusion with K^+^, as evidenced by a CPP of ~150 mmHg and a decrease in heart rate, could mimic ischemic preconditioning (Fig. S1D and G). However, short, repetitive I/R cycles are normally required for the induction of a cardioprotective effect. Interestingly, Okada *et al* (17) also discussed that DEX induced vasoconstriction may trigger ischemic preconditioning signalling as a possible mechanism of its cardioprotective effect. In summary, a protocol for ED induction without slight additional cardioprotective effects would be more meaningful to study effects on cardioprotection and an optimization of the protocol would be helpful.

Like pre-treatment, post-treatment with DEX also has no influence on IS or heart function regardless of presence of ED or physiological conditions. Furthermore, the DEX post-treatment attenuates improvement of LVDP by K^+^ after 60 min of reperfusion (Fig. S1C), which was observed in the group DexED. Negative effects induced by post-treatment with DEX on isolated perfused rat hearts using a constant flow Langendorff system were also reported by Mimuro *et al* (20). The authors measured an increase of infarct size compared with control hearts after I/R by applying 1 or 10 nM DEX immediately after ischemia with the onset of reperfusion. This increase was inhibited by yohimbine, indicating an α2-AR dependent mechanism.

In future studies, a possible inhibition of DEX-mediated cardioprotection by ED should be further investigated, as the existence of endothelial cells appears to be essential for successful cardioprotection by DEX. Riquelme *et al* (21) showed that IR-induced cardiomyocyte cell death could only be reduced by DEX when they were co-cultured with endothelial cells. Furthermore, these authors showed in isolated rat hearts that DEX activates endothelial nitric oxide (NO) synthase (eNOS) to produce NO using NOS inhibitor L-NAME (N-Nitroarginine methyl ester) or the NO scavenger PTIO (2-Phenyl-4,4,5,5-tetramethylimidazoline-1-oxyl 3-oxide). It should be taken into account that eNOS is not only present in endothelial cells, but also in cardiomyocytes. Therefore, cardiomyocytes could serve as additional source for NO production required for cardioprotection (22). A role for eNOS in cardioprotection against I/R injury by DEX preconditioning was also shown by Ibacache *et al* (23). In rat hearts pre-treated with 10 nM DEX for 25 min *in vivo* or *ex vivo*, phosphorylation of eNOS, extracellular signal-regulated kinase (ERK) 1/2 and Akt was increased. Furthermore, inhibition of the phosphatidylinositol 3-kinase (PI3K)/Akt/ERK/eNOS signalling cascade by the PI3K inhibitor LY-294002 abolished the infarct size-reducing effect of DEX preconditioning. To further unravel the signalling transduction of DEX preconditioning, this group analysed protein expression of the α2-AR isoforms A, B, C in extracts of whole adult rat hearts. All isoforms were detected, although the isotypes are differentially expressed in cardiomyocytes, endothelial cells and other cell types. Previously, Takahashi *et al* (18) analysed single cell RNA sequence data and showed that in human hearts all three isotypes are present, but none of them is predominantly expressed in cardiomyocytes. Whereas in endothelial cells, primarily α2-AR isoforms A and B are expressed (18). Therefore, the detailed interplay between cardiomyocytes and endothelial cells in the context of DEX preconditioning should be investigated in future studies to maintain the protective effect in the heart of patients with an ED. Not only PI3K/Akt/ERK/eNOS signalling but also other known mechanisms involved in the cardioprotective signalling of the cardioprotective effect of DEX preconditioning, such as the activation of large-conductance Ca^2+^-activated K^+^ channel or the involvement of miRNAs, could play a role here (7,18). Before further investigating the cardioprotective properties of DEX under ED, it is essential to establish a standardized cardioprotective protocol, defining the optimal concentration and treatment duration under constant flow conditions. Furthermore, translation to an *in vivo* model for I/R injury, for example left anterior descending occlusion in a mouse model, is imperative, as *ex vivo* models such as the Langendorff system do not fully reflect physiological conditions. This limitation is particularly relevant given that DEX also exerts effects via the central nervous system, which is not accounted for in the Langendorff system. Lastly, there are protocols for ED induction in mice, for example by chronic intraperitoneal administration of angiotensin II, allowing to study DEX preconditioning under ED *in vivo* (24).

A few limitations of the present study should be addressed. Additional modifications of the protocol for a successful cardioprotection by DEX were not implemented. For example, using higher concentrations of DEX or extending the perfusion time to 30 min could have led to a protective effect of DEX on IS and heart function. In addition, the slight protective effect by ED induction was not further analysed. For example, lower buffer temperatures, that is. hypothermia, could have contributed to this effect. Khaliulin *et al* (25) demonstrated that temperature conditioning is cardioprotective in isolated-perfused rat hearts by inhibition of opening probability of mitochondrial permeability-transition pore (mPTP) and improvement of heart function during reperfusion. A further limitation is the moderate standard deviation (SD) observed in infarct sizes. However, all groups exhibit a similar range of SDs regardless of treatment, suggesting that the variability reflects biological variation rather than treatment-specific effects. Another limitation of the present study is the exclusive use of male rats, even though this is the usual model in cardiovascular research. In the field of myocardial infarction and cardioprotection, numerous studies have already shown sex-specific differences (26). Sex-specific differences have also been identified for the effect of DEX. For example, Vincent *et al* (27) were able to show in female rats that the time to awaken from DEX anaesthesia varied depending on the female cycle, whereas awakening from anaesthesia with isoflurane, sevoflurane or propofol was independent of the female cycle. In humans, the sexes also appear to be differently susceptible to DEX, as a clinical study showed a significant dependence between sex and DEX dosage for sedation (28). Although these studies investigated the sedative properties of DEX with regard to sex-specific differences, it can be assumed that the cardioprotective or vasoconstrictive properties are also subject to such sex-specific influences. Thus, cardioprotective doses of DEX studied in male individuals should not be directly transferred to female individuals but should be explicitly analysed.

In conclusion, studies investigating cardioprotective effects of DEX should take into account its vasoconstrictive effect. Different hemodynamic conditions in *in vitro* models can modulate the cardioprotective properties of DEX.

## Supplementary Material

Hemodynamic variables. Quantification of hemodynamic variables for pre-treatment (yellow box) or post-treatment (blue box) with 3 nM Dex or vehicle (Con) under physiological conditions or under ED induced by perfusion with Krebs-Henseleit buffer containing 60 mM KCl (K+). (A) LVP max; (B) LVP min; (C) LVDP; (D) heart rate; (E) dP/dt max; (F) dP/dt min; (G) CPP. 1st TP: first treatment phase. Data are presented as the mean +/- SD. Statistical tests were only performed for baseline and at 60 min of reperfusion (Rep 60 min). One-way ANOVA followed by Tukey’s multiple comparisons tests for all comparisons at the respective time point. ^*^P<0.05 Dex vs. DexED (n=6-7). Dex, dexmedetomidine; ED, endothelial dysfunction; LVP max, maximal left ventricular pressure; LVP min, minimal LVP; LVDP, developed LVP; dP/dt max, maximal rate of rise of LVP; dP/dt min, minimal rate of rise of LVP; CPP, coronary perfusion pressure; ns, not significant (P>0.05).

## Figures and Tables

**Table I:** Peak pressure during ischemia (ischemia peak), body and heart weight. Data of experiments with a 5 min pre-treatment or a 10 min post-treatment (Post) with dexmedetomidine (DEX) or vehicle as control (Con) under physiological conditions or ED. Data are presented as the mean ± SD, n=6-7.

	Con	ConED	Dex	DexED	DexPost	DexPostED
Body weight, g	299±15	299±31	303±24	300±19	284±19	288±13
Ischemia peaks, mmHg	76±9	61±6^a^	76±7	62±9^b^	68±7	63±10
Heart weight wet, g	1.08±0.12	1.07±0.14	1.15±0.09	1.00±0.09	1.05±0.08	1.08±0.05
Heart weight dry, mg	138±17	150±20	144±9	145±30	137±8	147±14

One-way ANOVA followed by Tukey's multiple comparisons tests,

^a^P≤ 0.05 vs. Con;

^b^P≤0.05 vs. Dex. ED, endothelial dysfunction.

## Data Availability

The data generated in the present study may be requested from the corresponding author.

## References

[1] Heusch G (2023). Cardioprotection and its translation: A need for new paradigms? Or for new pragmatism? An opinionated retro- and perspective. J Cardiovasc Pharmacol Ther.

[2] Virani SS, Alonso A, Benjamin EJ, Bittencourt MS, Callaway CW, Carson AP, Chamberlain AM, Chang AR, Cheng S, Delling FN (2020). Heart disease and stroke statistics-2020 update: A report from the American heart association. Circulation.

[3] Kleinbongard P, Botker HE, Ovize M, Hausenloy DJ, Heusch G (2020). Co-morbidities and co-medications as confounders of cardioprotection-Does it matter in the clinical setting?. Br J Pharmacol.

[4] Segers VFM, Bringmans T, De Keulenaer GW (2023). Endothelial dysfunction at the cellular level in three dimensions: Severity, acuteness, and distribution. Am J Physiol Heart Circ Physiol.

[5] Roy R, Wilcox J, Webb AJ, O'Gallagher K (2023). Dysfunctional and dysregulated nitric oxide synthases in cardiovascular disease: Mechanisms and therapeutic potential. Int J Mol Sci.

[6] Cyr AR, Huckaby LV, Shiva SS, Zuckerbraun BS (2020). Nitric oxide and endothelial dysfunction. Crit Care Clin.

[7] Behmenburg F, Pickert E, Mathes A, Heinen A, Hollmann MW, Huhn R, Berger MM (2017). The cardioprotective effect of dexmedetomidine in rats is dose-dependent and mediated by BKCa channels. J Cardiovasc Pharmacol.

[8] Bunte S, Behmenburg F, Majewski N, Stroethoff M, Raupach A, Mathes A, Heinen A, Hollmann MW, Huhn R (2020). Characteristics of dexmedetomidine postconditioning in the field of myocardial ischemia-reperfusion injury. Anesth Analg.

[9] He L, Hao S, Wang Y, Yang W, Liu L, Chen H, Qian J (2019). Dexmedetomidine preconditioning attenuates ischemia/reperfusion injury in isolated rat hearts with endothelial dysfunction. Biomed Pharmacother.

[10] Torregroza C, Feige K, Schneider L, Bunte S, Stroethoff M, Heinen A, Hollmann MW, Huhn R, Raupach A (2020). Influence of hyperglycemia on dexmedetomidine-induced cardioprotection in the isolated perfused rat heart. J Clin Med.

[11] Castillo RL, Ibacache M, Cortinez I, Carrasco-Pozo C, Farías JG, Carrasco RA, Vargas-Errázuriz P, Ramos D, Benavente R, Torres DH, Méndez A (2019). Dexmedetomidine Improves Cardiovascular and ventilatory outcomes in critically Ill patients: Basic and clinical approaches. Front Pharmacol.

[12] Skrzypiec-Spring M, Grotthus B, Szelag A, Schulz R (2007). Isolated heart perfusion according to Langendorff-still viable in the new millennium. J Pharmacol Toxicol Methods.

[13] Cartier R, Pellerin M, Hollmann C, Pelletier LC (1993). Effects of pressure and duration of hyperkalemic infusions on endothelial function. Ann Thorac Surg.

[14] Raupach A, Karakurt E, Torregroza C, Bunte S, Feige K, Stroethoff M, Brandenburger T, Heinen A, Hollmann MW, Huhn R (2021). Dexmedetomidine provides cardioprotection during early or late reperfusion mediated by different mitochondrial K+-channels. Anesth Analg.

[15] Faul F, Erdfelder E, Lang AG, Buchner A (2007). G*Power 3: A flexible statistical power analysis program for the social, behavioral, and biomedical sciences. Behav Res Methods.

[16] Wang L, Liu J, Wang Z, Qian X, Zhao Y, Wang Q, Dai N, Xie Y, Zeng W, Yang W (2023). Dexmedetomidine abates myocardial ischemia reperfusion injury through inhibition of pyroptosis via regulation of miR-665/MEF2D/Nrf2 axis. Biomed Pharmacother.

[17] Okada H, Kurita T, Mochizuki T, Morita K, Sato S (2007). The cardioprotective effect of dexmedetomidine on global ischaemia in isolated rat hearts. Resuscitation.

[18] Takahashi K, Yoshikawa Y, Kanda M, Hirata N, Yamakage M (2023). Dexmedetomidine as a cardioprotective drug: A narrative review. J Anesth.

[19] Yoshikawa Y, Hirata N, Kawaguchi R, Tokinaga Y, Yamakage M (2018). Dexmedetomidine maintains its direct cardioprotective effect against ischemia/reperfusion injury in hypertensive hypertrophied myocardium. Anesth Analg.

[20] Mimuro S, Katoh T, Suzuki A, Yu S, Adachi YU, Uraoka M, Sano H, Sato S (2010). Deterioration of myocardial injury due to dexmedetomidine administration after myocardial ischaemia. Resuscitation.

[21] Riquelme JA, Westermeier F, Hall AR, Vicencio JM, Pedrozo Z, Ibacache M, Fuenzalida B, Sobrevia L, Davidson SM, Yellon DM (2016). Dexmedetomidine protects the heart against ischemia-reperfusion injury by an endothelial eNOS/NO dependent mechanism. Pharmacol Res.

[22] Ghasemi A, Jeddi S (2022). Quantitative aspects of nitric oxide production in the heart. Mol Biol Rep.

[23] Ibacache M, Sanchez G, Pedrozo Z, Galvez F, Humeres C, Echevarria G, Duaso J, Hassi M, Garcia L, Díaz-Araya G, Lavandero S (2012). Dexmedetomidine preconditioning activates pro-survival kinases and attenuates regional ischemia/reperfusion injury in rat heart. Biochim Biophys Acta.

[24] Trejo-Moreno C, Jimenez-Ferrer E, Castro-Martinez G, Méndez-Martínez M, Santana MA, Arrellín-Rosas G, Pedraza-Chaverri J, Medina-Campos ON, Hernández-Téllez B, Ramírez-Pliego O (2021). Characterization of a murine model of endothelial dysfunction induced by chronic intraperitoneal administration of angiotensin II. Sci Rep.

[25] Khaliulin I, Clarke SJ, Lin H, Parker J, Suleiman MS, Halestrap AP (2007). Temperature preconditioning of isolated rat hearts-a potent cardioprotective mechanism involving a reduction in oxidative stress and inhibition of the mitochondrial permeability transition pore. J Physiol.

[26] Querio G, Geddo F, Antoniotti S, Gallo MP, Penna C (2021). Sex and response to cardioprotective conditioning maneuvers. Front Physiol.

[27] Vincent KF, Mallari OG, Dillon EJ, Stewart VG, Cho AJ, Dong Y, Edlow AG, Ichinose F, Xie Z, Solt K (2023). Oestrous cycle affects emergence from anaesthesia with dexmedetomidine, but not propofol, isoflurane, or sevoflurane, in female rats. Br J Anaesth.

[28] Ding Y, Liu A, Wang Y, Zhao S, Huang S, Zhu H, Ma L, Han L, Shu S, Zheng L, Chen X (2023). Genetic polymorphisms are associated with individual susceptibility to dexmedetomidine. Front Genet.

