# Supplementary material for: Analysis of conditioning with dexmedetomidine under endothelial dysfunction in isolated perfused hearts
Source: Biomed Rep. 2025 Jun 11;23(2):136. doi: 10.3892/br.2025.2014 (PMC12207723; doi:10.3892/br.2025.2014)

Figure S1. Hemodynamic variables. Quantification of hemodynamic variables for pre-treatment (yellow box) or post-treatment (blue box) with 3 nM Dex or vehicle (Con) under physiological conditions or under ED induced by perfusion with Krebs-Henseleit buffer containing 60 mM KCl (K+). (A) LVP max; (B) LVP min; (C) LVDP; (D) heart rate; (E) dP/dt max; (F) dP/dt min; (G) CPP. 1st TP: first treatment phase. Data are presented as the mean +/- SD. Statistical tests were only performed for baseline and at 60 min of reperfusion (Rep 60 min). One-way ANOVA followed by Tukey's multiple comparisons tests for all comparisons at the respective time point. *P<0.05 Dex vs. DexED (n=6-7). Dex, dexmedetomidine; ED, endothelial dysfunction; LVP max, maximal left ventricular pressure; LVP min, minimal LVP; LVDP, developed LVP; dP/dt max, maximal rate of rise of LVP; dP/dt min, minimal rate of rise of LVP; CPP, coronary perfusion pressure; ns, not significant (P>0.05).

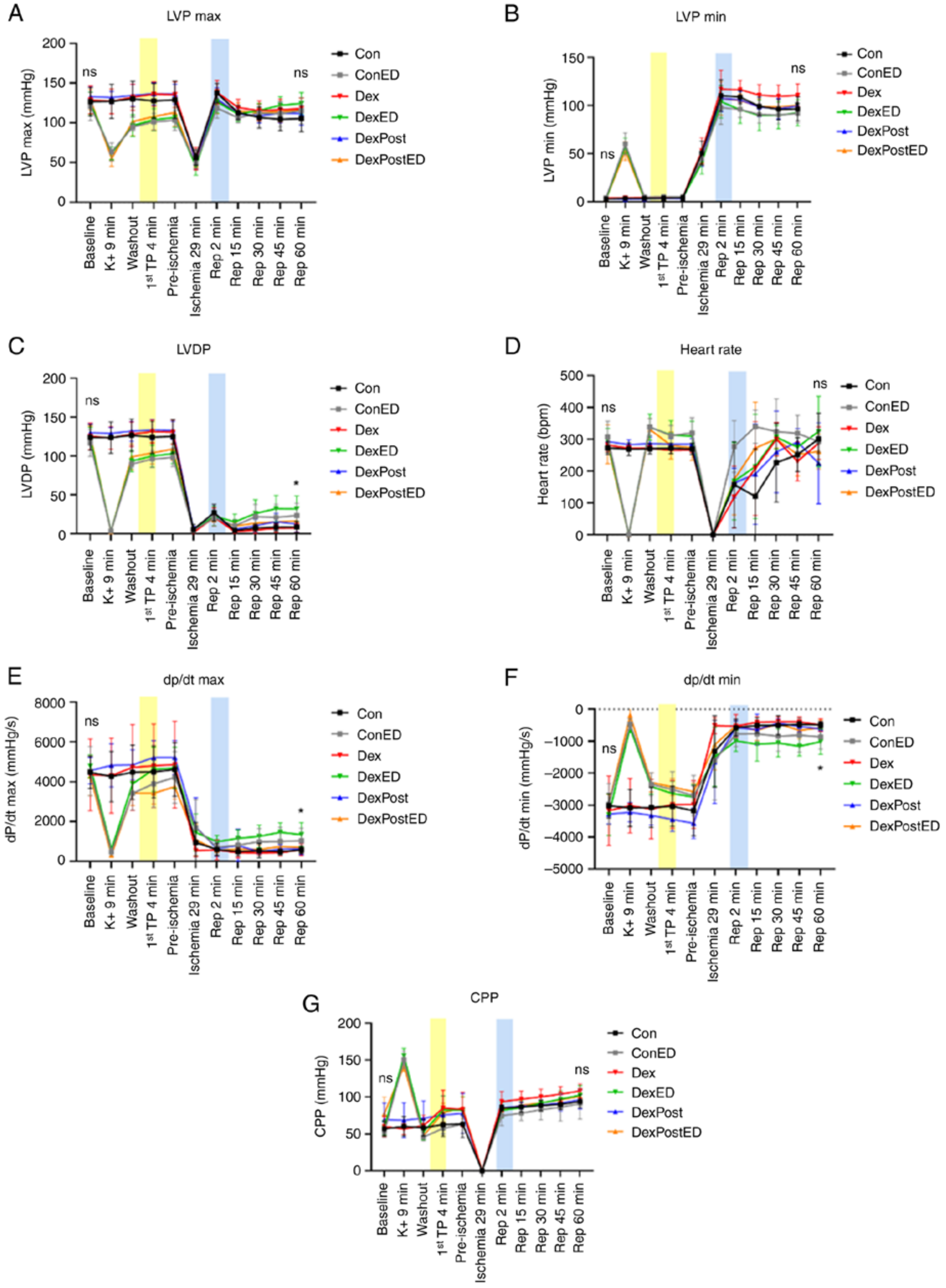

Supplement: Hemodynamic variables. Quantification of hemodynamic variables for pre-treatment (yellow box) or post-treatment (blue box) with 3 nM Dex or vehicle (Con) under physiological conditions or under ED induced by perfusion with Krebs-Henseleit buffer containing 60 mM KCl (K+). (A) LVP max; (B) LVP min; ( [file Supplementary_Data.pdf]
